# Hematological Profile of Non-infected Preterm Neonates in Eastern Morocco: A Retrospective Study at a Tertiary Care Center

**DOI:** 10.7759/cureus.114752

**Published:** 2026-08-18

**Authors:** Mahy Mohamed El Kerim, Mohammed Ech-Chebab, Anass Ayyad, Sahar Messaoudi, Rim Amrani

**Affiliations:** 1 Department of Neonatology and Neonatal Intensive Care Unit, Mohammed VI University Hospital, Oujda, MAR; 2 Mother and Child Health Laboratory, Mohammed 1st University, Oujda, MAR

**Keywords:** blood components, eastern morocco, neonatal hematology, preterm, reference values

## Abstract

Background

Preterm neonates are at increased risk of hematological abnormalities. However, region-specific reference values remain poorly defined in Morocco. This study aimed to provide descriptive regional data on hematological parameters in non-infected preterm neonates and to compare these parameters across three gestational age subgroups, namely, extremely preterm (<28 weeks), very preterm (28-31 weeks), and moderate preterm (32-36 weeks), admitted to a tertiary care center in Eastern Morocco.

Methodology

We conducted a retrospective descriptive study of preterm neonates (<37 weeks) admitted within the first 72 hours of life to the Neonatology and Neonatal Intensive Care Unit at Mohammed VI University Hospital, Oujda, between January and December 2023. Neonates with infection or significant maternal/perinatal conditions were excluded. Hematological parameters were analyzed according to gestational age group.

Results

A total of 112 non-infected preterm neonates were included. Statistically significant differences between gestational age groups were found for white blood cells (p = 0.009), neutrophils (p = 0.0004), mean corpuscular volume (p = 0.0006), and platelet counts (p = 0.022). Overall, hematological parameters were lower than those reported in international studies.

Conclusions

This study provides preliminary regional hematological data for preterm neonates. Differences from international values highlight the need for population-specific reference ranges.

## Introduction

Complete blood count (CBC) is a fundamental tool in neonatal care, providing essential information for diagnosis and management. These values change during pregnancy due to various maternal, environmental, and nutritional factors, as well as the degree of maturity of the newborns [1,2].

These premature newborns represent a fragile, vulnerable population with multiple hematological, infectious, and neurodevelopmental complications, with an overall incidence of 9.6% and an incidence ranging from 3% to 8% in Morocco [3,4]. Understanding their hematological profile is essential for early detection of pathological conditions and appropriate clinical management.

Preterm birth, defined as birth before 37 completed weeks of gestation, remains a leading cause of neonatal morbidity and mortality worldwide. According to Ohuma et al., an estimated 13.4 million preterm births occurred globally in 2020, representing approximately 10% of all live births [5]. In low- and middle-income countries, including Morocco, the burden is disproportionately higher, with national rates estimated between 3% and 8% and significant regional disparities [4,5].

Preterm neonates exhibit a distinct hematological profile that reflects the immaturity of their hematopoietic organs and the abrupt interruption of placental support. At birth, the transition from fetal to neonatal hematopoiesis results in marked changes in erythrocyte parameters, including elevated mean corpuscular volume (MCV) driven by the predominance of fetal hemoglobin, as well as labile leukocyte counts and thrombocytopenia, which are common complications of extreme prematurity [1,2]. These hematological abnormalities carry direct clinical implications, including increased transfusion requirements, susceptibility to nosocomial infections, and risk of intraventricular hemorrhage.

Reference hematological values for preterm neonates have been primarily established by large studies conducted in North American, European, Asian, and Latin American populations. Christensen et al. (2009) provided one of the most widely cited reference datasets, derived from over 47,000 neonates in a large multihospital system in the United States [1]. To date, no study has established baseline hematological reference values for preterm neonates in Morocco, particularly in the Eastern (Oriental) region. The absence of locally validated reference ranges may lead to misinterpretation of CBC results, with potential clinical consequences, including unnecessary therapeutic interventions or delayed recognition of true hematological disorders. This gap is particularly concerning given the documented impact of maternal nutritional status and the quality of prenatal care on fetal hematopoiesis in comparable Maghreb populations [4].

This study aims to describe the hematological profile of non-infected preterm neonates in our setting and compare it with international reference data.

## Materials and methods

Study design and period

This was a retrospective descriptive study based on the review of medical records of preterm neonates admitted to the Neonatology and Neonatal Intensive Care Unit of Mohammed VI University Hospital, a tertiary care center serving the Oriental region of Morocco. The study was conducted over a 12-month period, from January 1st to December 31st, 2023.

Patient recruitment

Inclusion Criteria

All preterm neonates meeting the following criteria were eligible for inclusion: (1) gestational age <37 completed weeks of gestation, confirmed by first-trimester obstetric ultrasound or by the Ballard/New Ballard clinical score; (2) admitted to the Neonatology and Neonatal Intensive Care Unit within the first 72 hours of life; and (3) no clinical or biological evidence of infection at the time of blood sampling. Infection was systematically ruled out based on all of the following: C-reactive protein <20 mg/L, procalcitonin <0.5 ng/mL, absence of pathological cerebrospinal fluid findings when lumbar puncture was performed, and negative urine culture.

Exclusion Criteria

The following neonates were excluded from the analysis: (1) those with confirmed neonatal infection or sepsis; (2) those born to mothers with significant perinatal complications likely to affect neonatal hematopoiesis, including chorioamnionitis, severe preeclampsia, or antepartum hemorrhage; (3) those with major congenital anomalies or chromosomal disorders; and (4) those with incomplete medical records.

Patient Flow

During the study period, a total of 192 preterm neonates were admitted to the Neonatology and Neonatal Intensive Care Unit. Of these, 167 (86.9%) were admitted within the first 72 hours of life. After applying the exclusion criteria, 112 non-infected preterm neonates were retained for final analysis. Neonates were categorized into three gestational age subgroups: extremely preterm (EP, <28 weeks, n = 10, 8.9%), very preterm (VP, 28-31 weeks, n = 48, 42.9%), and moderate preterm (MP, 32-36 weeks, n = 54, 48.2%).

Data collection

Data were extracted retrospectively from medical records using a standardized data collection form. Collected variables included gestational age (weeks), birth weight (g), sex, mode of delivery, maternal age, maternal comorbidities (diabetes, hypertension, anemia, chorioamnionitis), and exposure to antenatal corticosteroids. Blood samples were collected via umbilical catheter or peripheral venipuncture within the first 72 hours of life, as part of routine neonatal care. CBC was performed using the Sysmex XN-1000 automated hematology analyzer (Sysmex Corporation, Kobe, Japan), following standard laboratory operating procedures.

Outcomes

The primary outcome was the description and intergroup comparison of the following hematological parameters across the three gestational age subgroups: white blood cell (WBC) count, neutrophil count, lymphocyte count, monocyte count, red blood cell count (RBC), hemoglobin, hematocrit, MCV, mean corpuscular hemoglobin concentration (MCHC), and platelet count. The secondary outcome was the comparison of these values with published international reference data from studies conducted in France, the United States, Taiwan, South Korea, and Brazil.

Sample size calculation

This study adopted an exhaustive sampling approach, including all eligible neonates admitted during the 12-month study period.

Statistical analysis

Data were entered and analyzed using SPSS Statistics, version 27.0 (IBM Corp., Armonk, NY, USA). Continuous variables were expressed as mean ± standard deviation (SD), and categorical variables as absolute frequencies and percentages (n, %). The normality of the distribution of continuous hematological variables was assessed using the Shapiro-Wilk test. Given the non-normal distribution observed for most parameters, intergroup comparisons across the three gestational age subgroups (EP, VP, MP) were performed using the Kruskal-Wallis non-parametric test. A p-value <0.05 was considered statistically significant.

## Results

A total of 192 preterm neonates were admitted during the study period. Among them, 167 were admitted within the first 72 hours of life. After applying the exclusion criteria, 112 non-infected preterm neonates were included in the final analysis (Table 1).

**Table 1:** Demographic and clinical characteristics of the study population. M/F = male/female; MP = moderate preterm; VP = very preterm; EP = extremely preterm

Variable	Value
Total included neonates	112
Male sex (%)	62.5%
Sex ratio (M/F)	1.66
Mean birth weight (g)	1,907 (500–4,200)
MP (%)	48.2%
VP (%)	42.9%
EP (%)	8.9%
Mean maternal age (years)	28
Maternal diabetes (%)	8%
Maternal hypertension (%)	4.5%
Maternal anemia (%)	4.5%

The study population showed a male predominance (62.5%, n = 70), with a sex ratio of 1.66. The mean birth weight was 1,907 g (range = 500-4,200 g). A newborn whose mother had diabetes was included in the MP subgroup. The majority of neonates were MP (48.2%, n = 54), followed by VP (42.9%, n = 48) and EP (8.9%, n = 10).

Maternal characteristics revealed a mean age of 28 years. Diabetes was the most common maternal condition (8%), followed by anemia and hypertension (4.5%). Hematological parameters varied according to gestational age categories. Overall, MP neonates showed higher WBC compared to the VP and EP groups. Detailed hematological parameters are summarized in Tables 2-3.

**Table 2:** White blood cell parameters according to gestational age. Data are expressed as mean ± SD. Intergroup comparisons were performed using the Kruskal-Wallis test. WBC = white blood cell; EP = extremely preterm (<28 weeks); VP = very preterm (28–31 weeks); MP = moderate preterm (32–36 weeks)

Parameter	EP	VP	MP	H-statistic; p-value
WBC (×10³/µL)	11.54 ± 4.3	9.87 ± 5.06	12.58 ± 8.15	H = 9.40; p = 0.009
Neutrophils (×10³/µL)	4.35 ± 1.73	5.11 ± 3.37	7.43 ± 6.04	H = 15.55; p = 0.0004
Lymphocytes (×10³/µL)	4.38 ± 3.54	3.07 ± 1.68	3.01 ± 1.73	H = 1.53; p = 0.464
Monocytes (×10³/µL)	1.16 ± 0.55	1.22 ± 1.19	1.836 ± 1.14	H = 3.86; p = 0.145

**Table 3:** Red blood cell and platelet parameters according to gestational age. Data are expressed as mean ± SD. Intergroup comparisons were performed using the Kruskal-Wallis test. RBC = red blood cell; MCV = mean corpuscular volume; MCHC = mean corpuscular hemoglobin concentration; EP = extremely preterm (<28 weeks); VP = very preterm (28–31 weeks); MP = moderate preterm (32–36 weeks)

Parameter	EP	VP	MP	H-statistic; p-value
RBC (×10⁶/µL)	4.04 ± 0.74	4.37 ± 0.88	4.30 ± 0.62	H = 3.49; p = 0.175
Hemoglobin (g/dL)	15.5 ± 2.55	15.9 ± 2.96	16.1 ± 2.32	H = 1.30; p = 0.523
MCV (fL)	113 ± 9.65	106 ± 11.3	108 ± 7.48	H = 14.89; p = 0.0006
MCHC (g/dl)	32.9 ± 3.24	35.5 ± 3.28	34.9 ± 1.8	H = 1.22; p = 0.542
Hematocrit (%)	45.6 ± 7.13	45.9 ± 8.36	45.9 ± 6.72	H = 0.32; p = 0.853
Platelets (×10³/µL)	188.7 ± 93.7	189 ± 105	195 ± 89.1	H = 8.15; p = 0.017

## Discussion

Characteristics of the study population

Our study included 112 uninfected preterm infants hospitalized at the Mohammed VI University Hospital in Oujda during 2023. The distribution by degree of prematurity showed a predominance of moderate prematurity (MP, 32-36 weeks’ gestational age) at 48.2%(n = 54), followed by severe prematurity (VP, 28-31 weeks’ gestational age) at 42.9%(n = 48), and very severe prematurity (EP, <28 weeks’ gestational age) at 8.9% (n = 10). These proportions reflect the typical epidemiological distribution of prematurity described in the literature, where moderate-to-late prematurity accounts for the majority of preterm births [5].

A male predominance was observed (62.5%, n = 70), consistent with several international studies reporting a sex ratio slightly favoring boys among preterm infants [6]. Regarding maternal history, 67.85% of mothers had no significant history, while gestational diabetes was found in 8% of cases, which is likely to influence certain neonatal hematological parameters, particularly polycythemia [7]. These contextual data are important for interpreting the hematological values obtained.

Leukocyte

White Blood Cells and Neutrophils

In our series, the mean WBC counts were 11.54 ± 4.30 × 10³/μL for EP, 9.87 ± 5.06 × 10³/μL for VP, and 12.58 ± 8.15 × 10³/μL for MP. Statistical analysis using the Kruskal-Wallis test revealed a globally significant difference between the three groups (p = 0.009) (Table 4). These results are consistent with the data from Roudil et al. (France) [8], who reported in their study that all three blood cell lines increased proportionally with gestational age, confirming that the degree of prematurity is the main factor influencing blood count values at birth. In our study, the trend toward increased WBC counts with gestational age (from EP to MP) is consistent with this observation.

**Table 4:** Comparison of variations in white blood cell components across different studies. Data are expressed as mean ± SD. Intergroup comparisons were performed using the Kruskal-Wallis test. WBC = white blood cell; EP = extremely preterm (<28 weeks); VP = very preterm (28–31 weeks); MP = moderate preterm (32–36 weeks)

Parameter	Subgroup	Our study (2023)	Glasser et al. (2015) [18]	Roudil et al. (2017) [8]	Rolim et al. (2019) [14]	Bae et al. (2008) [12]	Wu et al. (2009) [11]
WBC (×10³/µL)	EP	11.54 ± 4.30	Not analyzed	9.28 ± 8.25	Not analyzed	11.04 ± 2.01	11.9 ± 9.81
	VP	9.87 ± 5.06	7.32	7.32 ± 2.83	Not analyzed	8.97 ± 1.84	10.58 ± 5.74
	MP	12.58 ± 8.15	10.37	12.66 ± 4.33	12.21 ± 4.32	6.81 ± 1.35	12.31 ± 4.67
Neutrophils (×10³/µL)	EP	4.35 ± 1.73	Not analyzed	3.64 ± 4.74	Not analyzed	3.92 ± 2.78	Not analyzed
	VP	5.11 ± 3.37	3.01	2.76 ± 1.8	Not analyzed	3.04 ± 2.63	Not analyzed
	MP	7.43 ± 6.04	4.91	6.94 ± 3.73	6.23 ± 3.34	3.07 ± 1.44	Not analyzed
Lymphocytes (×10³/µL)	EP	4.38 ± 3.54	Not analyzed	4.21 ± 2.35	Not analyzed	5.12 ± 2.14	Not analyzed
	VP	3.07 ± 1.68	3.23	3.65 ± 1.15	Not analyzed	4.41 ± 1.64	Not analyzed
	MP	3.01 ± 1.73	3.7	4.21 ± 1.56	4.96 ± 2.01	2.95 ± -0.89	Not analyzed
Monocytes (×10³/µL)	EP	1.16 ± 0.55	Not analyzed	0.75 ± 0.68	Not analyzed	0.59 ± 0.36	Not analyzed
	VP	1.22 ± 1.19	0.83	0.67 ± -0.49	Not analyzed	0.53 ± 0.33	Not analyzed
	MP	1.83 ± 1.14	1.16	1.16 ± 0.62	0.57	0.31 ± 0.19	Not analyzed

Compared to the study by Christensen et al. and the study by Jopling et al., our mean WBC values appear generally lower across the three subgroups [1,9]. Christensen et al. reported median leukocyte counts of approximately 12-15 × 10³/μL in preterm infants according to gestational age, values higher than ours, particularly for EP and VP. This difference could be explained by ethnic, environmental, and nutritional factors specific to our population, as well as by the quality of prenatal care, a factor recognized as a determinant of fetal hematopoietic maturation [10].

Regarding neutrophils, our mean values (4.35 ± 1.73, 5.11 ± 3.37, and 7.43 ± 6.04 × 10³/μL for EP, VP, and MP, respectively) showed a highly significant difference between groups (Kruskal-Wallis, p = 0.0004). The Taiwanese study by Wu et al. (2009) demonstrated that mode of delivery and gestational age significantly influenced polymorphonuclear neutrophil (PNN) values [11]. Our results confirm this relationship, with MP having higher PNN values than VP, consistent with the progressive maturation of granulopoiesis as gestational age advances.

Compared to the Korean study by Bae et al., the WBC and PNN values reported in this Asian population were also lower than ours for comparable stages of prematurity [12]. This observation aligns with those of other authors suggesting that ethnic and genetic differences in the regulation of granulopoiesis could explain interethnic variations in leukocyte reference values [10].

Lymphocytes and Monocytes

The mean lymphocyte counts in our study (4.38 ± 3.54, 3.07 ± 1.68, and 3.01 ± 1.73 × 10³/μL for EP, VP, and MP, respectively) did not show a statistically significant difference among the three groups (Kruskal-Wallis, p = 0.464) (Table 4). This result is consistent with data in the literature showing that lymphopoiesis in preterm infants is less directly dependent on gestational age than granulopoiesis [13]. Our values are close to those reported by Roudil et al. [8], who also found relative stability in lymphocyte counts regardless of the degree of prematurity. Similarly, the Brazilian study by Rolim et al. reported lymphocyte counts within a range similar to ours for MP [14]. Monocytes (1.16 ± 0.55 to 1.84 ± 1.14 × 10³/µL across subgroups) did not differ significantly between groups (p = 0.145), consistent with the data from Christensen et al., who also described a small variation in monocytes with gestational age [1].

Red blood cell lineage

Hemoglobin, Hematocrit, and Red Blood Cells

The mean hemoglobin values in our study were 15.5 ± 2.55 g/dL (EP), 15.9 ± 2.96 g/dL (VP), and 16.1 ± 2.32 g/dL (MP) (Table 5). No statistically significant differences were observed between the three groups (Kruskal-Wallis, p = 0.52), which could be explained by the physiological compensation between hepatic and splenic erythropoiesis, which is still active at these gestational ages, as well as by the persistence of elevated fetal hemoglobin, regardless of the degree of prematurity [15].

**Table 5:** Comparison of variations in red blood cell components (RBC, Hb, MCV, MCHC, Hct) across different studies. Data are expressed as mean ± SD where available, or as mean alone. Empty cells indicate data not reported; marked with "-". RBC = red blood cell count; Hb = hemoglobin; MCV = mean corpuscular volume; MCHC = mean corpuscular hemoglobin concentration; Hct = hematocrit; EP = extremely preterm; VP = very preterm; MP = moderate preterm

Parameter	Subgroup	Our study (2023)	Glasser et al. (2015) [18]	Roudil et al. (2017) [8]	Rolim et al. (2019) [14]	Bae et al. (2008) [12]	Wu et al. (2009) [11]
RBC (×10¹²/L)	EP	4.04 ± 0.74	Not analyzed	4.09 ± 0.46	Not analyzed	4.07 ± 0.44	3.91 ± 0.5
	VP	4.37 ± 0.88	3.94	4.24 ± 0.45	Not analyzed	4.24 ± 0.5	4.2 ± 0.39
	MP	4.3 ± 0.62	4.16	4.49 ± 0.60	4.36 ± 0.56	4.25 ± 0.44	4.35 ± 0.6
Hb (g/dL)	EP	15.5 ± 2.55	Not analyzed	15.54 ± 1.58	Not analyzed	15.52 ± 1.8	14.7 ± 1.62
	VP	15.9 ± 2.96	13.8	16.03 ± 1.88	Not analyzed	16.26 ± 1.91	15.6 ± 1.47
	MP	16.1 ± 2.32	14.3	16.44 ± 2.34	15.2 ± 2.1	16.86 ± 1.58	15.75 ± 2.15
MCV (fL)	EP	113 ± 9.65	Not analyzed	111.11 ± 6.84	Not analyzed	116.31 ± 7.62	115.4 ± 8.27
	VP	106 ± 11.3	110	107.87 ± 5.78	Not analyzed	114.36 ± 6.63	112.2 ± 6.7
	MP	108 ± 7.48	109.5	105.28 ± 5.86	108.1 ± 5.9	121.14 ± 7.81	108.25 ± 7.05
MCHC (g/dl)	EP	32.9 ± 3.24	Not analyzed	Not analyzed	Not analyzed	33.58 ± 1.17	Not analyzed
	VP	35.5 ± 3.28	31.85	Not analyzed	Not analyzed	33.67 ± 1.23	Not analyzed
	MP	34.9 ± 1.8	31.62	Not analyzed	33.1 ± 1.4	32.87 ± 1.67	Not analyzed
Hct (%)	EP	45.6 ± 7.13	Not analyzed	45.29 ± 4.24	Not analyzed	47.39 ± 5.78	44.8 ± 4.93
	VP	45.9 ± 8.36	43.2	45.76 ± 5.12	Not analyzed	48.35 ± 5.76	47.1 ± 3.96
	MP	45.9 ± 6.72	45.2	47.17 ± 6.35	47.0 ± 6.4	51.25 ± 3.56	46.75 ± 5.96

The study by Jopling et al. reported a median hemoglobin level of approximately 15.5-17.5 g/dL in preterm infants according to gestational age, values similar to ours [9]. Data from Roudil et al. also confirmed that hemoglobin at birth varied little with the degree of prematurity, unlike its postnatal evolution [8]. Hematocrit (45.75-46.62% in our series) was also not significantly different between groups (p = 0.85), consistent with the Taiwanese and Korean studies, which found similar values in Asian populations of preterm infants [11,12].

Mean Corpuscular Volume

MCV was the most discriminatory erythrocyte parameter in our study. We observed a significant trend toward a decrease in MCV as gestational age advanced: 113 ± 9.65 fL (EP), 106 ± 11.3 fL (VP), and 108 ± 7.48 fL (MP), with a highly significant difference between the groups (Kruskal-Wallis, p = 0.0006) (Table 5). This physiological macrocytosis, which is more pronounced in preterm infants with lower gestational age, is well documented in the literature and is explained by the predominance of fetal hemoglobin (HbF) in the RBCs of very preterm infants, and by the persistence of hepatic erythropoiesis producing larger-volume erythrocytes [15]. Roudil et al. confirmed that MCV was inversely correlated with gestational age, with very preterm infants exhibiting higher MCV values [8]. Our values (106-113 fL) are generally comparable to those reported by Christensen et al. and Wu et al. for comparable gestational ages [1,11].

Platelets

The mean platelet counts in our series were 188.7 ± 93.7 × 10³/μL (EP), 189 ± 105 × 10³/μL (VP), and 195 ± 89.1 × 10³/μL (MP). The Kruskal-Wallis test revealed a statistically significant difference between the groups (p = 0.022) (Table 6). MP infants had significantly higher platelet counts than very preterm infants. This result is consistent with data in the literature showing an increase in platelet production with the maturation of megakaryopoiesis, proportional to gestational age. Wiedmeier et al. established that normal platelet counts increase progressively with gestational age in preterm infants [16]. Our EP (188.7 ± 93.7 × 10³/μL) and VP (189 ± 105 × 10³/μL) values fall within the lower limit of normal defined by these reference studies, highlighting the higher susceptibility of very preterm infants to the so-called physiological thrombocytopenia of prematurity.

**Table 6:** Comparison of variations in platelets across different studies. Data are expressed as mean ± SD. Empty cells indicate data not reported by the study; marked with "-". EP = extremely preterm (<28 weeks); VP = very preterm (28–31 weeks); MP = moderate preterm (32–36 weeks).

Parameter	Subgroup	Our study (2023)	Glasser et al. (2015) [18]	Roudil et al. (2017) [8]	Rolim et al. (2019) [14]	Bae et al. (2008) [12]	Wu et al. (2009) [11]
Platelets (×10³/µL)	EP	188.7 ± 93.7	Not analyzed	193.88 ±51.49	Not analyzed	267.57 ± 89.78	243.5 ± 95.3
	VP	189.0 ± 105.0	153.5	210.35 ± 56.66	Not analyzed	261.96 ± 80.98	240.2 ± 59
	MP	195.0 ± 89.1	167.0	240.64 ± 61.76	293.11 ± 77.01	210.62 ±55.94	251.85 ± 73.3

The Brazilian study by Rolim et al. and the Taiwanese study by Wu et al. reported platelet counts higher than ours in comparable subgroups, which could reflect differences in the quality of maternal prenatal care, maternal nutritional status regarding iron and folate, factors whose impact on fetal megakaryopoiesis has been demonstrated [11,14,17].

Overall interpretation and factors explaining the observed differences

In general, the hematological values obtained in our population of preterm infants from the Oriental region are lower than those reported in American [18], Brazilian [14], French [8], Taiwanese [11], and Korean [12] studies for the majority of WBC and platelet parameters. Several non-mutually exclusive factors can be proposed to explain these differences.

First, maternal socioeconomic and nutritional factors: iron and folate deficiency, which is common in populations of eastern Morocco, as demonstrated by the study by Hassoune et al. in a comparable Maghreb population, is likely to impair fetal granulopoiesis and megakaryopoiesis [4].

Second, factors related to the quality of prenatal care: inadequate prenatal care, associated with unanticipated prematurity and reduced use of antenatal corticosteroid therapy, may influence the neonatal hematological profile. Antenatal corticosteroid therapy is known to induce transient leukocytosis, particularly neutrophilia, in the first days of life [19]. The heterogeneity of exposure to this therapy in our cohort constitutes a limitation that must be interpreted.

Third, ethnic and genetic differences in the regulation of hematopoiesis: several studies have demonstrated that genetic polymorphisms in genes regulating granulopoiesis and erythropoiesis lead to significant interethnic differences in reference values for blood counts, independent of any environmental factors [10,20].

Fourth, methodological differences: international reference studies have often been conducted with larger cohorts, allowing for fine stratification by gestational age (by week), whereas our study groups together the four- to nine-week gestational age range, which may dilute intragroup differences.

Limitations of the study

Our study had several limitations. First, the small sample size (112 cases) and the short duration (one year) do not allow for generalization of the results. Second, the heterogeneity of sampling methods (peripheral venous access and umbilical catheter), which can influence hematological values, as described in the literature. Finally, antenatal corticosteroid therapy was not considered in our study due to a lack of sufficient data to include it.

Outlook and clinical implications

Despite these limitations, our study provides the first baseline data on the hematological profile of uninfected preterm newborns in the Oriental region of Morocco. These data have several direct clinical implications: they allow for the local contextualization of neonatal blood count interpretation, help avoid the overdiagnosis of hematological disorders based on standards imported from non-comparable populations, and enable the adjustment of transfusion thresholds to local specificities.

Nationwide, multicenter prospective studies, with detailed stratification by gestational age, standardized sampling methods, and consideration of confounding variables (prenatal corticosteroid therapy, mode of delivery, prenatal care), are essential for establishing robust hematological reference values representative of the Moroccan population.

## Conclusions

This study provides the first preliminary descriptive hematological data for non-infected preterm neonates in Eastern Morocco. Statistically significant differences between gestational age subgroups were observed for WBC counts, neutrophils, MCV, and platelet counts. Overall values were lower than international references, likely reflecting ethnic, nutritional, and prenatal care factors specific to our population. Multicenter prospective studies are warranted to establish nationally validated reference ranges.
